# Trade in Cast-Off Teeth

**Published:** 1890-10

**Authors:** 


TRADE IN CAST-OFF TEETH.—A medical statistician estimates that the citizens of the United States are carrying gold to the value of £100,000 in the recesses of what ought to be their teeth. There are no people on the face of the globe who have such bad teeth and who spend so much money upon them as Americans, says a writer in the Popular Provider. No doubt the habit of hurried feeding and the wholesale consumption of sweet dishes have assisted much toward this end. But it is not a mistake to suppose, as says the medical statistician, that false teeth set in gold are buried when their owner shuffles off this mortal coil. If this is the custom in America it is not so in England, or why the numerous advertisements offering to buy old artificial teeth? The old teeth are not bought to use again, as some nervous people fancy, but simply for the sake of the gold.

